# Predicting prosthetic gait and the effects of induced stiff-knee gait

**DOI:** 10.1371/journal.pone.0314758

**Published:** 2025-01-02

**Authors:** Gilmar F. Santos, Eike Jakubowitz, Christof Hurschler

**Affiliations:** Laboratory for Biomechanics and Biomaterials, Department of Orthopedic Surgery, DIAKOVERE Annastift, Hannover Medical School, Hannover, Germany; Imperial College London, UNITED KINGDOM OF GREAT BRITAIN AND NORTHERN IRELAND

## Abstract

Prosthetic gait differs considerably from the unimpaired gait. Studying alterations in the gait patterns could help to understand different adaptation mechanisms adopted by these populations. This study investigated the effects of induced stiff-knee gait (SKG) on prosthetic and healthy gait patterns and the capabilities of predictive simulation. Self-selected speed gait of two participants was measured: one healthy subject and one knee disarticulation subject using a variable-damping microprocessor controlled knee prosthesis. Both performed unperturbed gait and gait with restricted knee flexion. Experimental joint angles and moments were computed using OpenSim and muscle activity was measured using surface electromyography (EMG). The differences between the conditions were analyzed using statistical parametric mapping (SPM). Predictive models based on optimal control were created to represent the participants. Additionally, a hypothetical unimpaired predictive model with the same anthropometric characteristics as the amputee was created. Some patterns observed in the experimental prosthetic gait were predicted by the models, including increased knee flexion moment on the contralateral side caused by SKG in both participants, which was statistically significant according to SPM. With the exception of the rectus femoris muscle, we also found overall good agreement between measured EMG and predicted muscle activation. We predicted more alterations in activation of the hip flexors than other muscle groups due to the amputation and in the activation of the biceps femoris short head, quadratus femoris, and tibialis anterior due to SKG. In summary, we demonstrated that the method applied in this study could predict gait alterations due to amputation of the lower limb or due to imposed SKG.

## Introduction

Lower extremity amputation can have different causes, such as trauma, tumor, and vascular diseases, including diabetes [1–3]. The specific prosthetic knee device employed in rehabilitation plays an important role in the quality of the gait of transfemoral amputees and knee disarticulation subjects. For example, microprocessor controlled knee (MPK) devices have been shown to provide stability in the absence of knee extensor muscles [4]. The development of innovative technologies has led to the design of various MPKs [5, 6]. In comparison to mechanically passive knee prostheses, MPKs have demonstrated improvement in gait symmetry, decreased metabolic rate, and enhanced smoothness of the gait [4, 7]. The Rheo Knee (Össur, Reykjavik, Iceland) is a variable-damping MPK that uses magnetorheological fluid to create knee flexion-resistance during the stance phase of gait [8]. Given the multitude of treatment options and prosthetic designs that is available, applying computational models to investigate how the prosthetic gait of an individual is affected by treatment options, such as prosthesis setup, could be of value in the rehabilitation process of lower limb amputees.

Recent advances in predictive simulation methods, in particular using optimal control, have shown promise in predicting new gait patterns independent of experimental data from motion capture devices or force plates. Thus, this type of musculoskeletal modeling may offer the opportunity to systematically investigate the cause-effect relationship of isolated interventions or pathologies that significantly affect the gait. Studies using optimal control with direct collocation have demonstrated the computational efficient prediction of unimpaired gait [9, 10]. Further studies have used optimal control to study the gait of transtibial amputees [11, 12]. However, a limitation of these studies was that the representation of prosthetic gait was two dimensional and restricted to the sagittal plane. Recent studies used optimal control and complex musculoskeletal models of patients with transfemoral amputation, demonstrating the feasibility of the method [13, 14]. Both studies performed tracking simulations, where the difference between the predicted gait pattern and an experimental result is minimized.

Despite its potential, the use of predictive simulation in clinically relevant studies has thus been limited to date [15, 16]. The validation and analysis of the feasibility of this method in different gait scenarios are still important steps in the direction of clinical relevance and application, since there is a dearth of literature on the use of fully predictive simulation. In a previous study, we were able to predict drop-foot gait in a post-stroke patient using a 3D musculoskeletal model [17]. The patient in that study also exhibited stiff-knee gait (SKG), which is a gait abnormality characterized by the lack of knee flexion during the swing phase of the gait. SKG is a common result of upper motor neuron injuries, observed for example after stroke, cerebral palsy, or spinal cord injury [18]. SKG is also often associated with over-activity of the rectus femoris muscle and other mechanisms [18, 19]. Healthy individuals using a knee orthosis that limits knee flexion may present alterations of intra-limb coordination similar to those of stroke patients with SKG [20].

In the current study, we chose to investigate the isolated effects of SKG, which was not possible in our previous study because the post-stroke patient investigated presented further complex gait abnormalities [17]. Instead, we chose to evaluate experimental data from two further individuals: one healthy subject and one knee disarticulation subject fitted with a variable-damping microprocessor knee prosthesis. The experimental data was used to calibrate the respective predictive models, and as ground-truth data to validate the predictions. Since the gait of the participants was captured under various conditions, such as unperturbed gait and gait with restricted knee flexion, it was possible to compare the experimental data and predictive results. This permitted the analysis of the predictive capabilities of the method. The general aim of this study was thus to investigate the feasibility of the method in a well-defined context, in order to investigate its potential for preliminary clinical use. The specific aims of this study were thus to predict prosthetic and healthy gait and the effects of induced SKG in two cases: a knee disarticulation subject fitted with a MPK, and a healthy subject walking with a knee orthosis. We hypothesized that an unimpaired predictive model, possessing the same anthropometric characteristics as the amputee, would accurately represent the pre-amputation healthy state of the patient. Thus, we created this model with the purpose of investigating the effect of different amputation parameters on the subject’s predicted amputated gait, such as the use of prosthetic foot and MPK, and altered muscle-tendon parameters.

## Materials and methods

### Experimental procedure

**Fig 1:**
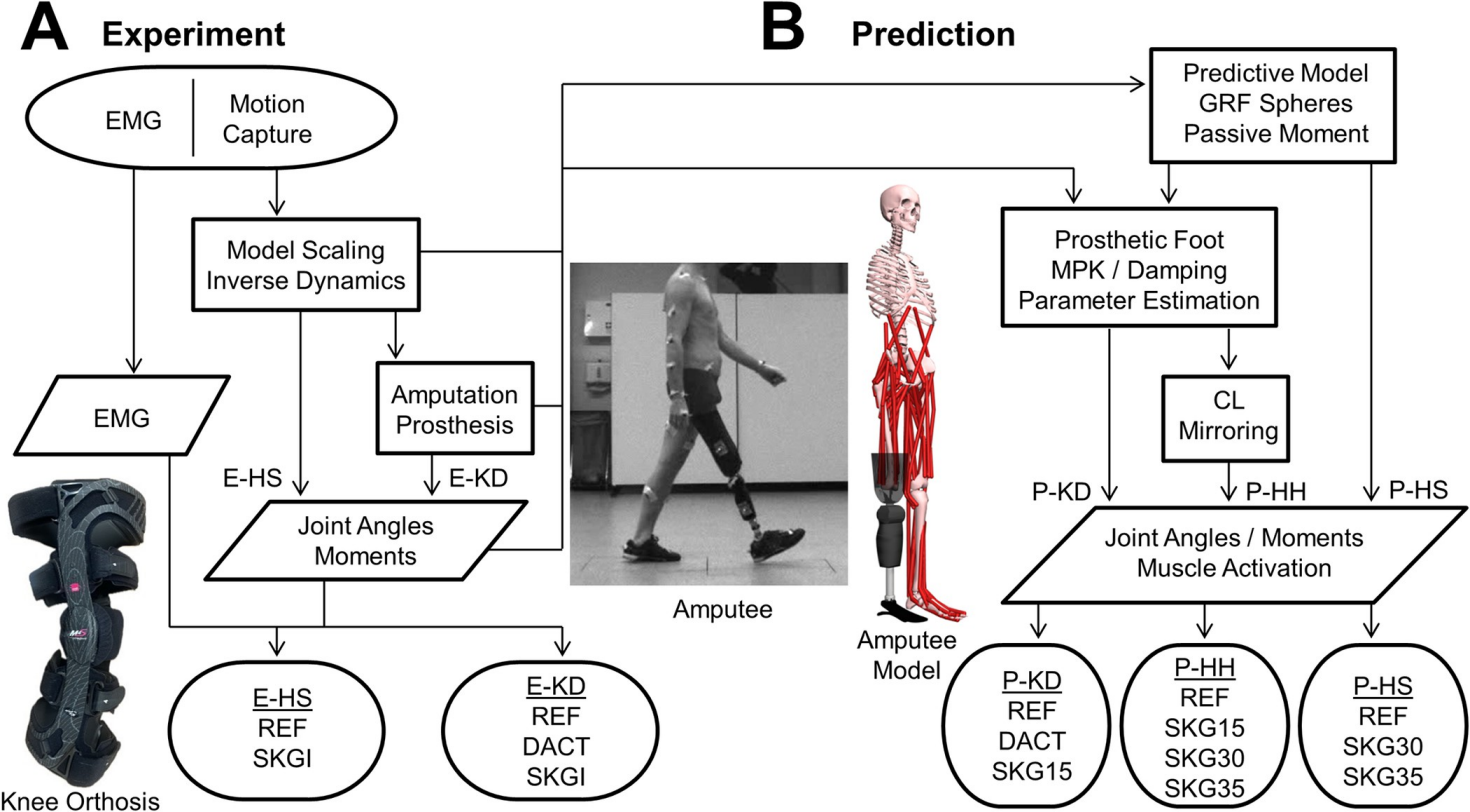
Flowchart of the experimental procedure and the predictive simulation. Experiment (A) and prediction (B).

Experimental motion capture data of a subject with unilateral knee disarticulation and of a healthy subject were collected (200 Hz) using two optical infrared systems: Vicon (MX, Vicon Motion System, Oxford, UK) and Qualisys (Göteborg, Sweden), respectively. Retroreflective markers were attached to the subjects in accordance to the Helen-Hayes marker set [21]. Ground reaction force (GRF) was measured (1000 Hz) using two force plates (type BP400600; AMTI, Watertown, MA, USA). The anthropometric data collected for each subject are reported in Table 1. Three conditions for the knee disarticulation subject (E-KD) and two conditions for the healthy subject (E-HS) were obtained (Table 1 and Fig 1A).

**Table 1:** Anthropometric characteristics and gait speed of the subjects and models.

	Subject	Condition	Gait speed (m·s^-1^)	Mass (kg)	Height (m)	Age (y)	Gender	Trials
**Experiment**	E-KD	REF	1.40 ± 0.02	68.5	1.78	43	Male	5
		DACT	1.46 ± 0.02					
		SKGI	1.34 ± 0.02					
	E-HS	REF	1.46 ± 0.03	77.8	1.81	26	Male	7
		SKGI	1.43 ± 0.04	78.4				
**Prediction**	P-KD	REF	1.48	68.5	1.78	−	−	−
		DACT						
		SKG15						
	P-HH	REF	1.48	72.1	1.78	−	−	−
		SKG15						
		SKG30						
		SKG35						
	P-HS	REF	1.48	77.8	1.81	−	−	−
		SKG30						
		SKG35						

Note: E-KD: experimental knee disarticulation; E-HS: experimental healthy subject; P-KD: predicted knee disarticulation, P-HH: hypothetical healthy predictive model of the amputee; P-HS: predicted healthy subject

The knee disarticulation subject walked at a self-selected speed using the MPK Rheo Knee and a carbon-fiber foot Vari-Flex (Össur, Reykjavik, Iceland). Three conditions were measured: using the optimal setting of the prosthesis (REF); with the prosthesis deactivated, which is a mode of operation used when the battery is discharged (DACT); and in a SKG induced condition, in which the prosthetic knee was locked to prevent flexion (SKGI). Since the patient wore a socket on the thigh, surface electromyography (EMG) data (Delsys, Natick, MA, USA) was collected (2000 Hz) only from the contralateral (CL) lower limb. Thus, 10 EMG electrodes were placed on CL muscles: gluteus maximus, gluteus medius, rectus femoris, vastus lateralis, vastus medialis, biceps femoris, semitendinosus, lateral gastrocnemius, medial gastrocnemius, and tibialis anterior. The data for E-KD was collected as part of a 5-year follow-up case study [22] and the data for our study was accessed on October 2021. We had access to information that could identify the participant during and after data collection.

The healthy subject walked at self-selected gait speed under two conditions: unperturbed gait (REF); and fitted with a knee orthosis (medi GmbH, Bayreuth, Germany) which induced SKG by constraining ipsilateral (IL) knee flexion to 20° (SKGI). In both conditions, EMG data was collected bilaterally: vastus lateralis, rectus femoris, biceps femoris, medial gastrocnemius, and tibialis anterior. Written informed consent to participate in the study was obtained, and the study was approved by the local Ethics Committee at Hannover Medical School (#10743). The recruitment period for this study was between March 2023 and May 2023.

The simulation software OpenSim (Version 3.3) was used to obtain the scaled anthropometric model, joint angles, and internal joint moments [23] of the two subjects respectively. The 3D lower body generic model gait2392, which consists of 23 degrees of freedom (DoF) and 92 muscles, was scaled to represent the anthropometric characteristics of the subjects. The metatarsophalangeal (MTP) joint was locked. For the E-KD model, the IL side was adapted to represent the amputation. The model of the MPK was designed and the other components were adapted from the literature [24] using an open-source computer-aided design (CAD) software (FreeCAD). The MPK and prosthetic foot consisted of one DoF each. For the E-HS model under the SKGI condition, the mass contribution of the knee orthosis was added to the IL femur and tibia segments.

### Predictive simulation

The predictive simulation of the gait and the parameter estimation of personalized muscle-tendon proprieties were formulated as optimal control problems by adapting the frameworks developed by Falisse et al. [10, 25]. The modeling procedure and choices were similar to those in previous work [17] and the flowchart of the predictive simulation is presented in Fig 1B. In order to solve the optimization problem, we used the direct collocation method with a third-order Radau collocation scheme to transcribe the problem into a nonlinear programming problem (NLP). CasADi is a framework that facilitates the implementation of numerical optimization [26]. It was used for the transcription of the optimal control problem into NLP. Since CasADi allows the application of algorithmic differentiation, the computational efficiency could be increased [10, 26]. The resulting optimization problem was solved using Interior Point Optimizer (IPOPT), which is a package that implements an interior point line search filter method to find a solution of large-scale nonlinear optimization problems [27]. The implementation of the frameworks was performed in MATLAB (R2019a, Mathworks, Natick, MA, USA).

The estimation of personalized muscle-tendon parameters was essential for the prediction of the prosthetic gait, in order to represent changes in the proprieties of muscles on the amputated side. The generic muscle-tendon parameters were obtained from the E-KD model after scaling in OpenSim. Since parameter estimation was performed only on the IL side, only 27 muscles that span the IL hip joint were included in the formulation of the optimal control problem (S1 Fig in S1 File). Optimal fiber length, tendon slack length, and maximal isometric force were personalized. The objective function that was minimized was defined according to Eq 1:

$$J_{Estim}=\int_{ti}^{tf}\sum \left(W_{E1}a^{2}+W_{E2}L_{opt}+W_{E3}{Re}^{2}+W_{E4}({\dot{a}}^{2}+{\dot{Fm}}^{2})\right)dt,$$ (1)

where *ti* and *tf* are the initial and final times, *W*_*E*1−4_ are the weight factors, *a* is the muscle activation, *L*_*opt*_ is the optimal fiber length, *Re* is the reserve actuator, and *Fm* is the tendon force. The number of mesh intervals was 100. The generic muscle-tendon parameters were used as the initial guesses, and the personalized parameters were limited to lie between 50% and 200% of the generic values. The predicted values are presented in S1 Fig in S1 File. The muscle redundancy problem was solved while the joint moments were reproduced [17, 25, 28].

We created a predictive knee disarticulation model (P-KD) to represent E-KD by adapting the musculoskeletal model used to obtain the experimental results. Six contact spheres were included to model the foot-ground interaction represented as a Hunt-Crossley contact [23, 29]. The values for the GRF sphere parameters were calibrated for the patient [10, 17]. The MTP joint of the CL foot was unlocked and a passive moment, a linear rotational spring, and a damper were included [30]. The passive moment, which represents the passive structures of the joint, was also included in all joints using an exponential function (S2 Fig in S1 File). Thus, when the joint angle exceeded a certain limit, a passive resistive moment in the opposite direction was created [17]. We chose passive moment parameters of the prosthetic foot to reproduce the characteristics of energy storage and return (ESR) during gait. The model of the MPK included a passive moment to avoid hyperextension (S2 Fig in S1 File), as well as activation dynamics that consisted of MPK excitation and activation. A damper was also included in the formulation of the MPK to replicate the damping effect of the device. The function in Eq 2 was used to describe the damper:

$$T_{MPK}=a_{MPK}D_{MPK}\dot{q},$$ (2)

where *T*_*MPK*_ is the moment created on the prosthesis, *a*_*MPK*_ is the activation term, *D*_*MPK*_ is the damper coefficient, and $\dot{q}$ is the angular velocity of the prosthetic knee. The only difference between the REF and DACT conditions for P-KD was the value of the damping coefficient *D*_*MPK*_. In REF, we used the value of 1 N·m·s·rad^-1^ while in the DACT 0.75 N·m·s·rad^-1^ was used. These values were chosen empirically. In the SKG15 condition, the flexion angle limit of the passive moment in the MPK used in the REF condition was reduced from 137.5° to 14.9°, thus constraining MPK flexion (S2 Fig in S1 File).

The states of the P-KD were positions and velocities of the DoF, muscle activations, muscle-tendon forces, and activation of the prosthetic knee. The controls were the time derivative of the states and MPK excitation. The objective function used for P-KD is described by Eq 3:

$$J_{P-KD}=\int_{ti}^{tf}\sum \left(W_{P1}a^{2}+W_{P2}{\dot{E}}^{2}+W_{P3}{\ddot{q}}^{2}+W_{P4}({\dot{a}}^{2}+{\dot{Fm}}^{2})+W_{P5}{e_{MPK}}^{2}\right)\frac{1}{Dist}dt,$$ (3)

where *M*_*P*1−5_ are the weight factors (S1 Table in S1 File), $\dot{E}$ is the metabolic energy rate, $\ddot{q}$ is the joint acceleration, *e*_*MPK*_ is the prosthetic knee excitation, and *Dist* is the distance traveled by the pelvis in the forward direction. Periodicity and gait speed were imposed, but the time of the complete gait cycle was a variable of the system. The number of mesh intervals used in the gait predictions was 400. One trial of the E-KD in the DACT condition was used to obtain the initial guess, bounds and scaling for joint kinematics for all conditions of P-KD (S3 Fig in S1 File). To analyze the effect of altering the initial guess, further simulations were performed using a different trial to create a second initial guess (IG2), which are presented only in S3 Fig in S1 File.

The muscle-tendon model used was the Hill-type model [28, 31] and the muscle activation dynamics were described using Raasch’s model [32, 33]. Polynomial functions of joint positions and velocities were used to define muscle-tendon lengths, velocities and moment arms [10, 34]. The personalized muscle-tendon parameters obtained from the parameter estimation were used on the IL side while the generic parameters where used on the CL side.

We mirrored the CL side of P-KD in order to create a hypothetical healthy predictive model (P-HH) to represent the healthy state of the knee disarticulation. Since intact limbs weigh more than the prosthesis, the total mass of the patient was increased (Table 1). Four conditions were simulated: REF, in which the P-HH model was symmetric; SKG15, in which the IL knee flexion angle limit of the passive moment was reduced from 137.5° to 14.9°; SKG30, in which the angle limit was 30°; and SKG35, in which the angle limit was 35° (S2 Fig in S1 File). The initial guess and bounds of the joint kinematics were based on the gait data of a healthy subject from Falisse et al. [10]. The formulations of P-HH and P-KD were basically the same, with the exceptions of the initial guess, and the prosthesis and the related excitation term of the MPK in the objective function in Eq 3, which were removed. We also created a predictive model based on the E-HS model (P-HS), which represented the anthropometric characteristics of the healthy subject. Three conditions of the P-HS were predicted: the REF, SKG30, and SKG35 conditions. The differences between P-HH and P-HS were the weight factors of objective function, the values of the GRF sphere parameters, and the body scaled model. The weight factors used in the objective functions are presented in S1 Table in S1 File. The same gait speed, which was the fastest speed observed in experimental data, was imposed in all the predictive models and conditions (Table 1). Thus, the influence of gait speed was removed from the predictions. The computational time of the gait predictive simulations, which were run on a standard laptop (Intel Core i7-1260P), and other information about the convergence of the optimization problem are presented in S2 Table in S1 File.

### Data analysis

The differences between the conditions in the experimental results were analyzed using statistical parametric mapping (SPM) [35]. We used an open-source one-dimensional SPM package (SPM1D, www.spm1d.org) in MATLAB to compare joint angles, moments, and EMG results in different conditions of gait for E-KD and E-HS. The paired t-test was used with a critical threshold of 5% (α = 0.05). A bar below the graphs indicates a statistically significant difference based on SPM. Due to the relatively small number of trials in our data (5 and 7 trials), we were not able to perform a test for normality using SPM, which requires at least 8 trials. Pataky et al. suggested that a comparison between the parametric and non-parametric results should be performed in this case [36]. We compared both procedures and some of the results diverged (S4 Fig in S1 File). Thus, we considered our data non-Gaussian and the non-parametric procedure was chosen [36, 37].

Since we obtained only one result from each prediction and not repeated trials as in the experimental data, SPM could not be used for comparisons of P-KD, P-HS, and P-HH. We thus used dynamic time warping (DTW) to analyze the similarities between the predicted muscle activation patterns and between mean experimental and predicted gait parameters, such as sagittal joint angles and moments. DTW allows shifting during the measurement of distance between two time series. This feature allows a better analysis of the shape of the curves that are not aligned, which can occur in time series data [38]. A DTW score for each comparison was obtained in MATLAB whereby a value of zero would indicate that the curves are identical. The shifting was restricted to a maximum of 5%. The gait parameters were normalized by dividing the curves by the absolute maximum value in the data set [39]. Thus, DTW was performed in the normalized series (values within the range of -1 to 1).

## Results

### Effects of MPK damping on prosthetic gait

**Fig 2:**
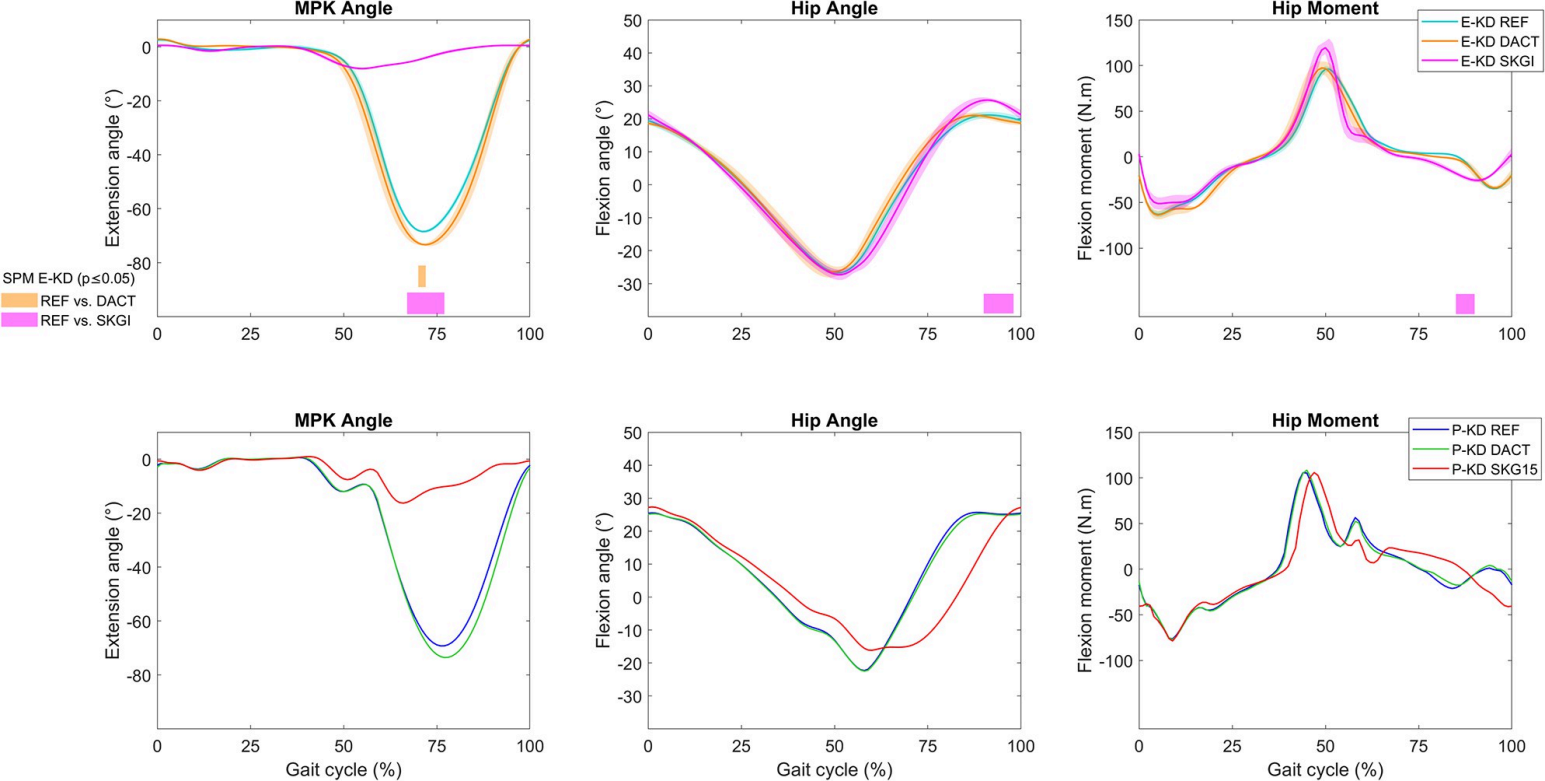
Kinematics and kinetics on ipsilateral (IL) side of the knee disarticulation subject. MPK sagittal angle, and hip sagittal angle and moment for E-KD (top panels) and P-KD (bottom panels), and SPM analysis of E-KD for REF vs. DACT and REF vs. SKGI (bars below the graphs). The MPK passive moment is presented in S2 Fig in S1 File. Detailed SPM analysis is presented in S4 Fig in S1 File.

Peak experimental (E-KD) MPK flexion angle in the swing phase increased from 68.4° in the REF condition to 73.4° in the DACT condition (Fig 2). This increase was significant only at about 70% of the gait cycle. The same effect was observed for peak predicted (P-KD) MPK flexion in the swing phase, which similarly increased from 69.2° to 73.5°. The simulations using a different initial guess also showed an increased MPK flexion during DACT in comparison to REF (S3 Fig in S1 File). No significant differences in the IL hip angle and moment were observed in E-KD between REF and DACT. In the prediction (P-KD), the change in damping also did not considerably affect hip angle or moment (Fig 2).

### Effects of SKG on prosthetic gait

Peak experimental (E-KD) MPK flexion was reduced to 8.1°, and peak predicted (P-KD) knee flexion to 16.3° in SKG conditions (Fig 2). This decrease in SKGI in comparison to REF observed in E-KD was significant according to SPM. A detailed SPM analysis is presented in S4 Fig in S1 File. We observed a significant increase in IL hip flexion angle and differences in hip moment in E-KD SKGI at the end of the gait cycle. In P-KD REF, we predicted patterns of IL hip angle and moment that were similar to those in E-KD REF, but the deviations caused by SKG15 did not correspond to the experimental results (Fig 2).

**Fig 3:**
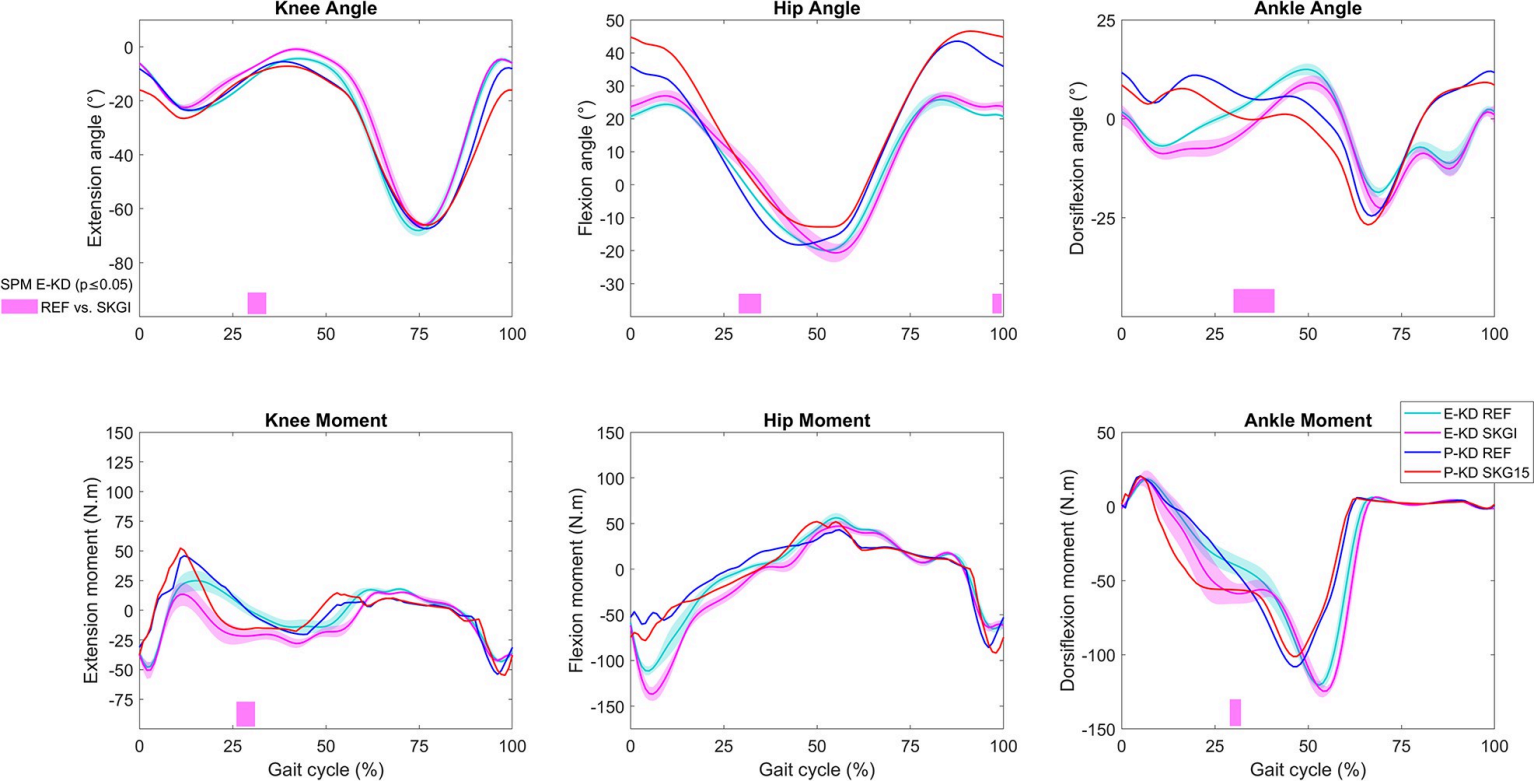
Kinematics and kinetics on contralateral (CL) side of the knee disarticulation subject. Knee, hip and ankle sagittal angles (top panels) and moments (bottom panels) for E-KD and P-KD, and SPM analysis of E-KD for REF vs. SKGI (bars below the graphs).

In general, CL kinematics and kinetics showed good agreement between experimental (E-KD) and predicted (P-KD) values, with exception of the hip and ankle angles at the beginning and end of the gait cycle, *i*.*e*., during swing-to-stance transition (Fig 3 and S5 Fig in S1 File). CL sagittal knee and ankle angles were significantly affected in the stance phase by the lack of IL MPK flexion under SKG conditions. While the experimental knee angle pattern was similar to the prediction, we observed increased hip flexion and ankle dorsiflexion angle in the P-KD relative to those observed in E-KD (Fig 3). Experimental CL hip flexion, knee extension, and ankle plantarflexion angle, and knee flexion and ankle plantarflexion moments were all significantly increased during SKGI. The predicted CL joint moment patterns were similar to E-KD and the effects of SKGI were predicted in P-KD SKG15 (Fig 3). The increase in CL knee flexion moment was also predicted using a different initial guess (S3 Fig in S1 File).

**Fig 4:**
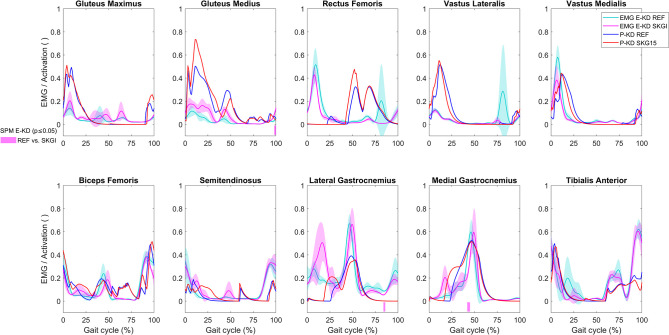
EMG and muscle activation on contralateral (CL) side of the knee disarticulation subject. EMG for E-KD, muscle activation for P-KD, and SPM analysis of E-KD for REF vs. SKGI (bars below the graphs). The predicted muscle activation curves depicted in the graph of some muscles are composed of the maximal values between their components as follows: biceps femoris composed of biceps femoris long and short heads; gluteus maximus composed of gluteus maximus 1, 2, and 3; and gluteus medius composed of gluteus medius 1, 2, and 3.

Only an increase of gluteus medius EMG at the end of the gait cycle, a decrease of lateral gastrocnemius at 85% of the gait cycle, and a delayed peak of medial gastrocnemius EMG at 43% of the gait cycle were significantly different between SKGI and REF of E-KD (Fig 4). At about 20% of the gait cycle, increased gluteus medius EMG was observed and also predicted. While an advanced offset of EMG in SKGI was observed in rectus femoris and vastus medialis, the P-KD model predicted the same changes in activation of the vastus lateralis and medialis muscles. The model did not predict the first peak of rectus femoris activity. An increase of rectus femoris activation was predicted during SKG15 in the middle of the gait cycle, but not observed in the EMG.

From about 10% to 30% of the gait cycle, SKGI caused an increase of EMG of the knee flexor muscles (biceps femoris, semitendinosus, and lateral and medial gastrocnemius) (Fig 4). While not statistically significant, this increase was predicted (P-KD) in the same muscles between from about 15% to 40% of the gait cycle. We observed a slightly delayed peak of lateral and medial gastrocnemius EMG activity during SKGI, which was also predicted for muscle activation.

### Effects of SKG on unimpaired gait

**Fig 5:**
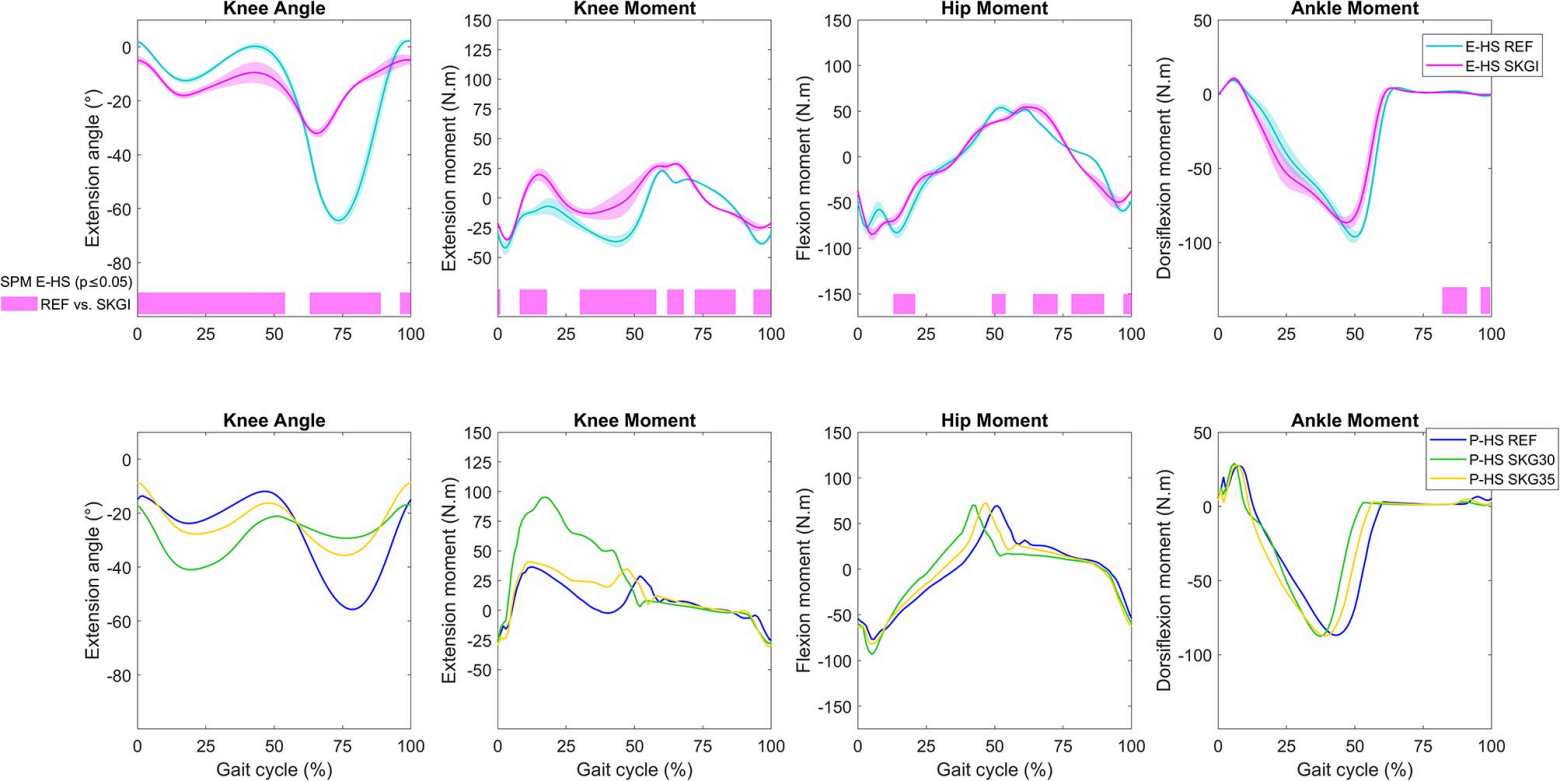
Kinematics and kinetics on ipsilateral (IL) side of the healthy subject. Knee sagittal angle and moment and hip and ankle sagittal moments for E-HS (top panels) and P-HS (bottom panels), and SPM analysis of E-HS for REF vs. SKGI (bars below the graphs). Kinematics of hip and ankle joints and contralateral joint moments are presented in S7 and S6 Figs in S1 File, respectively. Ipsilateral knee sagittal passive moment is presented in S2 Fig in S1 File.

The use of the knee orthosis caused significant differences on the IL side in E-HS during the entire gait cycle (Fig 5). In the stance phase, we observed increased knee flexion angle and extension moment in the experimental results of SKGI. During the swing phase, the peak of knee flexion was decreased, as expected. P-HS REF predicted less IL knee range of motion than E-HS REF. However, hip and ankle moments exhibited higher similarity than other gait parameters in both sides comparing E-HS and P-HS in REF (S5 Fig in S1 File). Both SKG conditions predicted increased knee flexion angle in the stance phase. Even though knee flexion was more constrained in SKG30 than in SKG35, SKG30 predicted more knee flexion in the stance phase than SKG35 in P-HS. In the swing phase, however, the decrease of peak knee flexion was consistent with the limitations imposed on the prediction (Fig 5). Both SKG conditions predicted increased knee extension moment, which was more evident in SKG30 than in SKG35 because of the larger passive moment (S2 Fig in S1 File). While we observed a statistically significant delay of the peak of IL hip flexion moment in E-HS SKGI, P-HS predicted an earlier peak in both SKG30 and SKG35. No significant difference was observed in experimental ankle moment in the stance phase, while an advanced peak of ankle plantarflexion moment was predicted in SKG30 and SKG35. On the CL side, significant differences in E-HS were observed mostly at about 30% of the gait cycle, and these differences were predicted (S6 and S7 Figs in S1 File).

**Fig 6:**
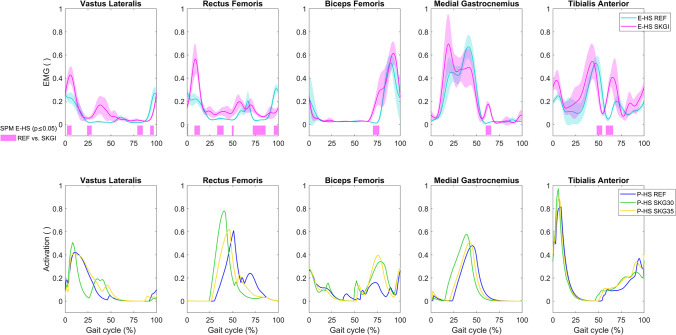
EMG and muscle activation on ipsilateral (IL) side of the healthy subject. EMG for E-HS (top panels), muscle activation for P-HS (bottom panels), and SPM analysis of E-HS for REF vs. SKGI (bar below the graph). Corresponding contralateral curves are presented in S8 Fig in S1 File. The predicted muscle activation curves depicted in the graph of biceps femoris are composed of the maximal values between biceps femoris long and short heads.

Statistically significant differences between REF and SKGI were observed in the IL EMG of all muscles. The EMG patterns of vastus lateralis and rectus femoris, which are knee extensors, were similar in E-HS (Fig 6). A significant increase in peak activity at the beginning of the gait cycle, increases between 25% and 80% of the gait cycle, and a decrease at the end were observed in the EMG of these muscles during SKGI. Once again, the peak activity of the knee extensors of E-HS at the beginning of the gait cycle was predicted only in the vastus lateralis of P-HS. The increase in peak of vastus lateralis muscle activity was predicted only in SKG30, while the other aforementioned effects were predicted in both SKG30 and SKG35. A statistically significant increase in biceps femoris activity at 75% of the gait cycle was observed in the EMG of E-HS SKGI and predicted in P-HS SKG30 and SKG35. The increase in medial gastrocnemius activity at 63% of the gait cycle was not predicted. However, the advanced onset in medial gastrocnemius activity, which was not statistically significant, and the increase in tibialis anterior EMG of E-HS during SKGI were predicted under SKG conditions. On the CL side, significant differences between REF and SKGI were observed in vastus lateralis, rectus femoris, biceps femoris, and tibialis anterior EMG of E-HS, and P-HS predicted more deviations in rectus femoris activation in SKG30 and SKG35 compared with REF (S8 Fig in S1 File).

### Prediction of a hypothetical unimpaired gait

**Fig 7:**
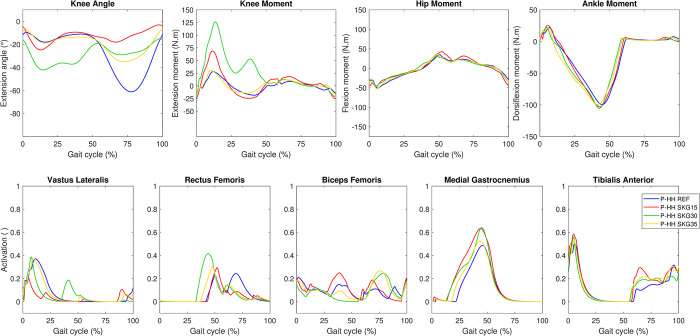
Kinematics, kinetics and muscle activation on ipsilateral (IL) side of the hypothetical healthy predictive model of the amputee. Knee sagittal angle and moment, hip and ankle sagittal moments (top panels), and muscle activation (bottom panels) for P-HH. Corresponding contralateral curves are presented in S9 Fig in S1 File. Ipsilateral knee sagittal passive moment is presented in S2 Fig in S1 File. The predicted muscle activation curves depicted in the graph of biceps femoris are composed of the maximal values between biceps femoris long and short heads.

In general, the gait pattern of the hypothetical healthy predictive model (P-HH) was similar to the unimpaired gait in the REF condition (Fig 7 and S5 Fig in S1 File). The effects of SKG30 and SKG35 on IL knee angle of P-HH were similar to those of P-HS. In SKG15, where knee flexion was constrained the most, we observed an intermediate peak of knee flexion angle and extension moment during the stance phase in relation to SKG30 and SKG35. This is related to the implementation of the passive moment (S2 Fig in S1 File). We observed the lowest peak of knee flexion angle during the swing phase in SKG15. SKG conditions affected IL hip and ankle moment in comparison to REF to a lesser extent than observed in the knee joint and caused different patterns on IL muscle activation. The peak and advanced onset of medial gastrocnemius and tibialis anterior activation observed in SKG15, SKG30, and SKG35 were greater than those observed for REF (Fig 7). The CL gait pattern of P-HH shows that we could predict a symmetric gait in REF and that the SKG conditions affected the CL side differently resulting in asymmetric gait (S9 Fig in S1 File).

### Prediction of muscle activation: P-KD vs. P-HH

**Fig 8:**
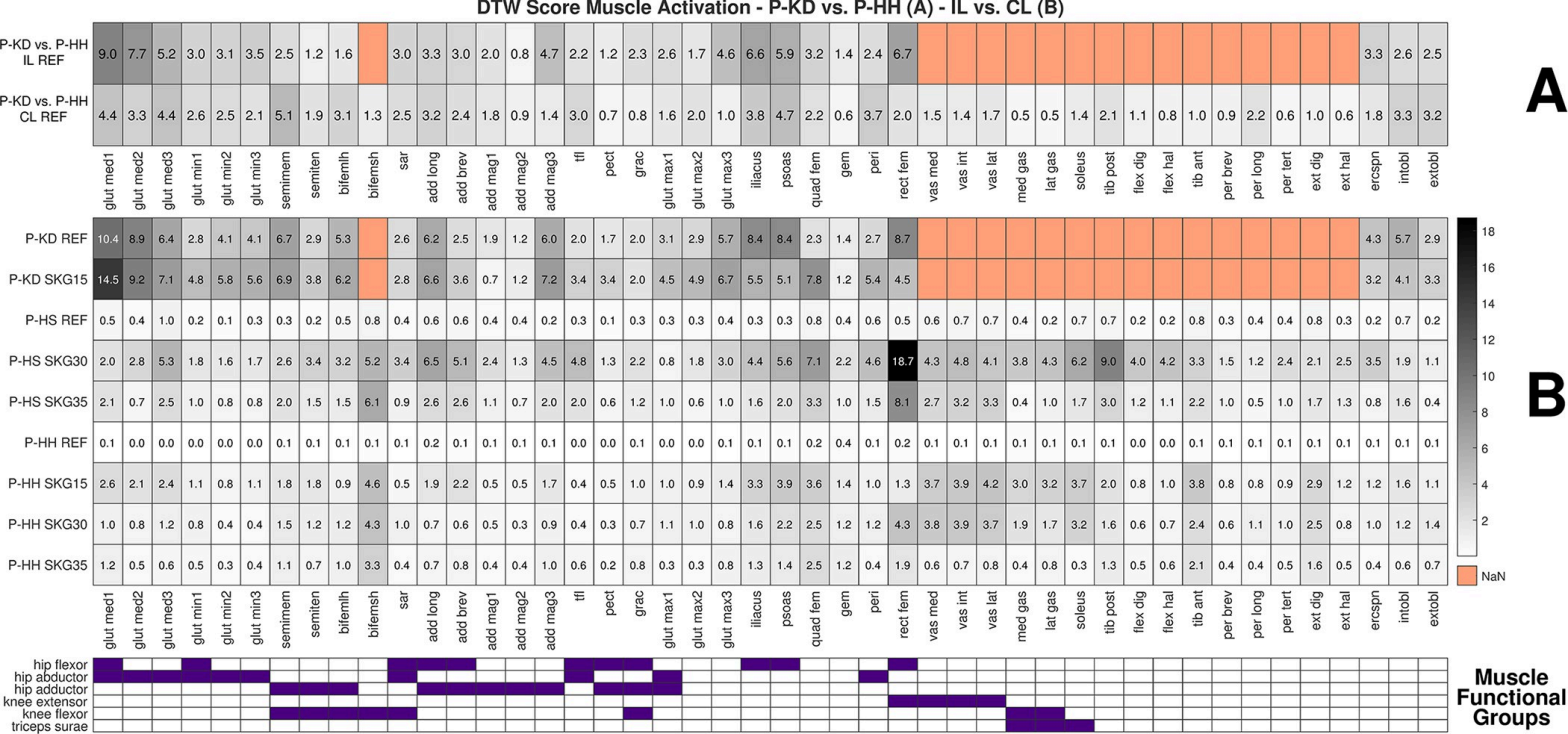
Heat map of DTW scores of the similarity in predicted muscle activations. Comparison of the REF condition of P-KD vs. P-HH (A) and the ipsilateral (IL) vs. contralateral (CL) side of several models (B). Smaller (lighter) DTW values indicate greater agreement; higher (darker) DTW values indicate lesser agreement between the curves. Not a number (NaN) was assigned to the muscles that are non-existent on the IL side of P-KD. Functional groups of some muscles are depicted at the bottom. The full name of the muscles and their functional groups are presented in S3 Table in S1 File.

Since P-KD (amputee) and P-HH (hypothetical healthy) represent the same patient, comparing the similarities of predicted muscle activation between these models facilitated the investigation of which muscles were more affected by amputation. Comparing the muscle activation of the IL side in the REF condition of P-KD to P-HH, we observed larger differences in gluteus medius, rectus femoris, and iliopsoas muscles than in the remaining muscles (Fig 8A). Here, all the muscles with increased differences in activation act as hip flexors. Furthermore, comparing the CL with the IL side, we further observed a clear increase in the DTW score on the CL side in several muscles: semimembranosus, biceps femoris long head, tensor fasciae latae, and piriformis, all of which act as hip abductors or adductors (Fig 8A).

### Prediction of muscle activation: Symmetry

In order to analyze the symmetry of predicted muscle activation, we compared the IL side to the CL side of the same model and condition (Fig 8B). P-KD predicted asymmetrical muscle patterns both in the REF and SKG15 conditions. SKG15 increased asymmetry compared with REF, except in the iliopsoas and rectus femoris muscles (Fig 8B). Both P-HS and P-HH predicted nearly symmetrical muscle patterns in REF. However, the introduction of various limitations on knee flexion (SKG conditions) increased asymmetry in different muscles between models. We observed that the biceps femoris short head, quadratus femoris, and tibialis anterior muscles were affected the most by SKG in both P-HS and P-HH models. The knee extensor muscles were clearly asymmetrical in P-HS SKG30 and SKG35 and in P-HH SKG30, while the gluteus medius, iliopsoas, vasti, and triceps surae muscles exhibited higher asymmetry in the more constrained condition of each model, i.e., P-HS SKG30 and P-HH SKG15 (Fig 8B).

## Discussion

The objectives of this study were to predict prosthetic and healthy gait under different conditions and to validate the predicted results against the experimental data. We have shown that in the context of the knee disarticulation, predictive simulation allowed the generation of new motions independent of experimental data. The predictions presented similar characteristics to the experimental data. For instance, we observed in the experimental results a subtle increase of MPK flexion when the patient walked with the prosthetic knee deactivated (Fig 2). We could predict a similar result when only the damping was altered in the predictive model, which indicates that we could model the dynamic response of the device. This complex prosthetic modeling along with the muscle-tendon parameter estimation and the application a fully predictive simulation could be considered improvements in comparison to the literature, since we did not track the experimental results [13, 14]. Moreover, we also observed an increased peak of IL hip flexion moment on the prosthetic gait in comparison to the CL side and to the unimpaired gait (Fig 2). We observed this effect in our predictive models and also in the hypothetical unimpaired model. We could also predict to a certain degree the effects of SKGI on CL joint moments, which were similar between E-KD and E-HS (Fig 3 and S6 and S9 Figs in S1 File). This suggests that the cause-effect relationships of certain changes on the musculoskeletal system, such as amputation and stiff-knee gait, can be predicted using this method, even though the prediction of ankle angle differed from the experimental data (S5 and S7 Figs in S1 File).

Knee disarticulation has been performed since 1581, but it is less common than transfemoral amputation, even though they might have similar functional outcomes [40, 41]. Due to the limited literature on knee disarticulation, we decided to also compare our results to studies performed in transfemoral amputees. We do not intent with this to draw generic conclusions, but it is important to analyze whether our results are in accordance with the literature. The gait of transfemoral amputees may be affected by muscle strength deficits [42]. An MRI study has shown that the muscles of the stump from transfemoral amputees suffered atrophy differently, with the gluteus maximus and quadriceps femoris exhibiting greater changes in comparison to the intact side, the adductor muscles suffering less atrophy, and the adductor longus not being atrophied [43]. Since these effects are not homogeneous, it is important that the predictive model can account for differences in muscle properties in order to better represent the patient. To this end, we personalized the muscle properties of the knee disarticulation subject by performing a parameter estimation procedure to personalize muscle-tendon properties of the IL side. In our personalized model, the maximal isometric force of the adductor brevis and magnus and gluteus maximus muscles was reduced (S1 Fig in S1 File). However, these results should be interpreted carefully as optimal fiber length and tendon slack length, two other parameters describing the muscles, were also altered. In general, these parameters are difficult to measure and they might affect the estimation of muscle forces [17, 44].

SKGI was modeled using large knee passive moments (S2 Fig in S1 File). In SKG15 and SKG30 conditions of P-HH and P-HS, respectively, we observed increased IL knee flexion angle and knee passive extension moment in the stance phase, but decreased knee extensor muscle activation (Figs 5–7 and S2 Fig in S1 File). However, in P-KD, which does not have muscles spanning the knee joint on the IL side, this effect was not observed. Previous work has shown that during predictive simulation, the passive moment may be exploited to reduce muscle effort [17, 45], which is also the case in the current study. Further investigation is needed to analyze whether this approach accurately represents the effects of using a knee orthosis.

A peak in the measured rectus femoris EMG was observed for both E-KD and E-HS during swing-to-stance transition. However, this peak was not predicted. A study that measured surface and fine wired EMG of the rectus femoris reported that surface EMG is subjected to cross-talk with the vastus intermedius [46], which may partly explain the differences in muscle activity we observed between the experimental EMG and predicted muscle activation. With this exception, we found overall good agreement between predicted activation and measured EMG. A study that used SPM vector-field analysis could identify differences that are not observed by means of independent scalar analyses of EMG data [47]. This could explain why some effects of SKGI on EMG were not statistically significant according to SPM, but were predicted. Since our work focused on the validation of the predicted results, we decided to analyze and compare individual muscles rather than compare group of muscles.

Our predictive results showed that amputation affected more clearly the hip flexor muscles on the IL side, which is consistent with a study that observed high activation of hip flexor muscles in bilateral transfemoral and through-knee persons [48]. Differences in activation of the gluteus medius and iliopsoas were observed on both sides, but more so for the gluteus medius on the IL side (Fig 8A). This is in agreement with the literature, where this muscle also exhibited differences in EMG between controls and transfemoral amputees [49]. We could predict nearly symmetric muscle patterns in P-HH and P-HS REF as demonstrated by very similar muscle activation of the IL and CL sides in the respective models (Fig 8B). The asymmetry observed in the predictions was not intuitive, since SKG15, SKG30, and SKG35 affected the muscles differently in P-KD, P-HH, and P-HS. For example, in all results we observed a subtle increase in biceps femoris activation and EMG at 20% of the gait cycle on the CL side. However, for medial gastrocnemius, a similar increase was observed only in E-KD, P-KD, and P-HH, even though both muscles are knee flexors (Fig 4 and S8 and S9 Figs in S1 File). This suggests that the models could predict some responses observed in experimental data, even though P-KD SKG15 differed more from the experimental results than other conditions, especially for IL hip angle (Fig 2 and S5 Fig in S1 File). The possible reasons for this deviation could be that the implementation of SKG15 in P-KD did not represent accurately the locked MPK in E-KD SKGI. Furthermore, the simulations did not converge when MPK flexion angle was more limited than 14.9°, which was the case during E-KD SKGI.

A limitation of our work is that for technical reasons, we were not able to measure EMG and muscle-tendon properties of the stump, and so we were not able to validate the muscle activation of the amputated side in our predictions. Accessing the muscles on the side of the prosthesis is difficult without altering the socket to facilitate EMG electrodes, which was not an option in our case. Another limitation is that we present the results of only two subjects. Therefore, our study is merely a proof of concept and our findings cannot be generalized to different type of amputations and other individuals.

The gait pattern predicted using optimal control represents one possible solution for the optimization problem, so we cannot insure that the global minimum was found. To address this limitation of optimal control particularly for the knee disarticulation patient, we tested different initial guesses (S3 Fig in S1 File). Similarly to Miller et al. [13], some of these simulations did not converge. This made this process challenging, because the gait simulations converged between 1 and 13 hours (S2 Table in S1 File) and if the prediction of one gait condition did not converge, the other results were not used. Changing the initial guess yielded different costs of the objective function (S2 Table in S1 File) and slightly differences in the gait patterns, which did not affected our analysis, since the main characteristics of the prosthetic gait and effects of the gait conditions were the same as presented (S3 Fig in S1 File). However, in the current study we cannot draw conclusions about the correlation between these differences caused by altering the initial guess and variations observed when a participant walks repeatedly under the same condition. Even though one trial of the experimental results was used as initial guess, the prediction may still be considered independent of the experimental data, since we did not minimize the differences between experimental and predicted results. Furthermore, the same initial guess was used for all gait conditions and the alterations of parameters of the system predicted different results.

## Conclusion

In conclusion, we predicted the prosthetic gait of a knee disarticulation subject, including the effects of different prosthetic knee settings. Moreover, we created a hypothetical model of the patient that predicted an optimal gait pattern of the amputee without impairment which was similar to that of a healthy subject. Thus, we were able to investigate which muscles in the predictions were affected by amputation and also by the imposed restrictions of knee flexion in the respective models. In this work, we showed that predictive simulation using optimal control can be a feasible approach to predict changes in gait due to musculoskeletal deficits such as lower limb amputation or imposition of SKG. Future work could focus on applying these methods to a greater number of subjects, improving prosthesis modeling, and investigating the effects of SKG in other clinical contexts.

## Supporting information

S1 File Additional data. File containing additional tables and figures.
(PDF)

## References

[1] Hammarlund CS, Carlström M, Melchior R, Persson BM. Prevalence of back pain, its effect on functional ability and health-related quality of life in lower limb amputees secondary to trauma or tumour: a comparison across three levels of amputation. Prosthet Orthot Int. 2011 Mar;35(1):97–105. doi: 10.1177/0309364610389357 21515895

[2] Moxey PW, Gogalniceanu P, Hinchliffe RJ, Loftus IM, Jones KJ, Thompson MM, et al. Lower extremity amputations—a review of global variability in incidence. Diabet Med. 2011 Oct;28(10):1144–53. doi: 10.1111/j.1464-5491.2011.03279.x 21388445

[3] Sawers AB, Hafner BJ. Outcomes associated with the use of microprocessor-controlled prosthetic knees among individuals with unilateral transfemoral limb loss: a systematic review. J Rehabil Res Dev. 2013;50(3):273–314. doi: 10.1682/jrrd.2011.10.0187 23881757

[4] Kaufman KR, Frittoli S, Frigo CA. Gait asymmetry of transfemoral amputees using mechanical and microprocessor-controlled prosthetic knees. Clinical Biomechanics. 2012 Jun;27(5):460–5. doi: 10.1016/j.clinbiomech.2011.11.011 22221344 PMC3335968

[5] Bellmann M, Schmalz T, Blumentritt S. Comparative biomechanical analysis of current microprocessor-controlled prosthetic knee joints. Arch Phys Med Rehabil. 2010 Apr;91(4):644–52. doi: 10.1016/j.apmr.2009.12.014 20382300

[6] Thiele J, Schöllig C, Bellmann M, Kraft M. Designs and performance of three new microprocessor-controlled knee joints. Biomed Tech (Berl). 2019 Feb 25;64(1):119–26. doi: 10.1515/bmt-2017-0053 29425102

[7] Johansson JL, Sherrill DM, Riley PO, Bonato P, Herr H. A clinical comparison of variable-damping and mechanically passive prosthetic knee devices. American Journal of Physical Medicine & Rehabilitation. 2005 Aug;84(8):563–75. doi: 10.1097/01.phm.0000174665.74933.0b 16034225

[8] Herr H, Wilkenfeld A. User‐adaptive control of a magnetorheological prosthetic knee. Industrial Robot. 2003 Feb;30(1):42–55. doi: 10.1108/01439910310457706

[9] Ackermann M, van den Bogert AJ. Optimality principles for model-based prediction of human gait. J Biomech. 2010 Apr 19;43(6):1055–60. doi: 10.1016/j.jbiomech.2009.12.012 20074736 PMC2849893

[10] Falisse A, Serrancolí G, Dembia CL, Gillis J, Jonkers I, De Groote F. Rapid predictive simulations with complex musculoskeletal models suggest that diverse healthy and pathological human gaits can emerge from similar control strategies. J R Soc Interface. 2019 Aug 30;16(157):20190402. doi: 10.1098/rsif.2019.0402 31431186 PMC6731507

[11] Koelewijn AD, van den Bogert AJ. Joint contact forces can be reduced by improving joint moment symmetry in below-knee amputee gait simulations. Gait Posture. 2016 Sep;49:219–25. doi: 10.1016/j.gaitpost.2016.07.007 27459416

[12] Price MA, Umberger BR, Sup FC. Dynamic optimization of gait with a generalized lower-limb prosthesis model. In: 2019 IEEE 16th International Conference on Rehabilitation Robotics (ICORR) [Internet]. Toronto, ON, Canada: IEEE; 2019 [cited 2020 Dec 3]. p. 734–9. Available from: https://ieeexplore.ieee.org/document/8779532/10.1109/ICORR.2019.8779532 31374718

[13] Miller RH, Bell EM, Russell Esposito E. Transfemoral limb loss modestly increases the metabolic cost of optimal control simulations of walking. PeerJ. 2024;12:e16756. doi: 10.7717/peerj.16756 38223753 PMC10785795

[14] Vandenberg NW, Wheatley BB, Carpenter RD, Christiansen CL, Stoneback JW, Gaffney BMM. Feasibility of predicting changes in gait biomechanics following muscle strength perturbations using optimal control in patients with transfemoral amputation. Comput Methods Biomech Biomed Engin. 2024 Sep 10;1–15. doi: 10.1080/10255842.2024.2399038 39256913 PMC11891085

[15] De Groote F, Falisse A. Perspective on musculoskeletal modelling and predictive simulations of human movement to assess the neuromechanics of gait. Proc R Soc B. 2021 Mar 10;288(1946):20202432. doi: 10.1098/rspb.2020.2432 33653141 PMC7935082

[16] Fregly BJ. A conceptual blueprint for making neuromusculoskeletal models clinically useful. Appl Sci. 2021 Mar;11(5):2037. doi: 10.3390/app11052037

[17] Santos GF, Jakubowitz E, Pronost N, Bonis T, Hurschler C. Predictive simulation of post-stroke gait with functional electrical stimulation. Sci Rep. 2021 Nov 1;11(1):21351. doi: 10.1038/s41598-021-00658-z 34725376 PMC8560756

[18] Tok F, Balaban B, Yaşar E, Alaca R, Tan AK. The effects of onabotulinum toxin A injection into rectus femoris muscle in hemiplegic stroke patients with stiff-knee gait: a placebo-controlled, nonrandomized trial. Am J Phys Med Rehabil. 2012 Apr;91(4):321–6. doi: 10.1097/PHM.0b013e3182465feb 22311056

[19] Campanini I, Merlo A, Damiano B. A method to differentiate the causes of stiff-knee gait in stroke patients. Gait Posture. 2013 Jun;38(2):165–9. doi: 10.1016/j.gaitpost.2013.05.003 23755883

[20] Celestino ML, van Emmerik R, Barela JA, Bacca O, Barela AMF. Effects of limited knee flexion movement in intra-limb gait coordination. J Biomech. 2021 Nov 9;128:110712. doi: 10.1016/j.jbiomech.2021.110712 34474372

[21] Kadaba MP, Ramakrishnan HK, Wootten ME. Measurement of lower extremity kinematics during level walking. J Orthop Res. 1990 May;8(3):383–92. doi: 10.1002/jor.1100080310 2324857

[22] Einfeldt AK, Yao D, Daniilidis K, Calliess T, Welke B, Jakubowitz E. Knee disarticulation following oncologic total knee arthroplasty: A 5-year follow-up case report. Clin Biomech. 2022 Mar 1;94:105608. doi: 10.1016/j.clinbiomech.2022.105608 35248833

[23] Delp SL, Anderson FC, Arnold AS, Loan P, Habib A, John CT, et al. OpenSim: open-source software to create and analyze dynamic simulations of movement. IEEE Trans Biomed Eng. 2007 Nov;54(11):1940–50. doi: 10.1109/TBME.2007.901024 18018689

[24] LaPrè AK, Price MA, Wedge RD, Umberger BR, Sup FC. Approach for gait analysis in persons with limb loss including residuum and prosthesis socket dynamics. Int J Numer Meth Biomed Engng [Internet]. 2018 Apr [cited 2020 Dec 2];34(4). doi: 10.1002/cnm.2936 29111608

[25] Falisse A, Pitto L, Kainz H, Hoang H, Wesseling M, Van Rossom S, et al. Physics-based simulations to predict the differential effects of motor control and musculoskeletal deficits on gait dysfunction in cerebral palsy: A retrospective case study. Front Hum Neurosci. 2020;14:40. doi: 10.3389/fnhum.2020.00040 32132911 PMC7040166

[26] Andersson JAE, Gillis J, Horn G, Rawlings JB, Diehl M. CasADi: a software framework for nonlinear optimization and optimal control. Math Prog Comp. 2019 Mar;11(1):1–36. doi: 10.1007/s12532-018-0139-4

[27] Wächter A, Biegler LT. On the implementation of an interior-point filter line-search algorithm for large-scale nonlinear programming. Math Program. 2006 May;106(1):25–57. doi: 10.1007/s10107-004-0559-y

[28] De Groote F, Kinney AL, Rao AV, Fregly BJ. Evaluation of direct collocation optimal control problem formulations for solving the muscle redundancy problem. Ann Biomed Eng. 2016 Oct;44(10):2922–36. doi: 10.1007/s10439-016-1591-9 27001399 PMC5043004

[29] Sherman MA, Seth A, Delp SL. Simbody: multibody dynamics for biomedical research. Procedia IUTAM. 2011;2:241–61. doi: 10.1016/j.piutam.2011.04.023 25866705 PMC4390141

[30] Falisse A, Afschrift M, De Groote F. Modeling toes contributes to realistic stance knee mechanics in three-dimensional predictive simulations of walking. PLoS One. 2022;17(1):e0256311. doi: 10.1371/journal.pone.0256311 35077455 PMC8789163

[31] Muscle Zajac F. and tendon: properties, models, scaling, and application to biomechanics and motor control. Crit Rev Biomed Eng. 1989;17(4):359–411. 2676342

[32] De Groote F, Pipeleers G, Jonkers I, Demeulenaere B, Patten C, Swevers J, et al. A physiology based inverse dynamic analysis of human gait: potential and perspectives. Comput Methods Biomech Biomed Eng. 2009 Oct;12(5):563–74. doi: 10.1080/10255840902788587 19319704

[33] Raasch CC, Zajac FE, Ma B, Levine WS. Muscle coordination of maximum-speed pedaling. J Biomech. 1997 Jun;30(6):595–602. doi: 10.1016/s0021-9290(96)00188-1 9165393

[34] van den Bogert AJ, Geijtenbeek T, Even-Zohar O, Steenbrink F, Hardin EC. A real-time system for biomechanical analysis of human movement and muscle function. Med Biol Eng Comput. 2013 Oct;51(10):1069–77. doi: 10.1007/s11517-013-1076-z 23884905 PMC3751375

[35] Pataky TC. One-dimensional statistical parametric mapping in Python. Comput Methods Biomech Biomed Engin. 2012;15(3):295–301. doi: 10.1080/10255842.2010.527837 21756121

[36] Pataky TC, Vanrenterghem J, Robinson MA. Zero- vs. one-dimensional, parametric vs. non-parametric, and confidence interval vs. hypothesis testing procedures in one-dimensional biomechanical trajectory analysis. J Biomech. 2015 May 1;48(7):1277–85. doi: 10.1016/j.jbiomech.2015.02.051 25817475

[37] Nichols TE, Holmes AP. Nonparametric permutation tests for functional neuroimaging: a primer with examples. Hum Brain Mapp. 2002 Jan;15(1):1–25. doi: 10.1002/hbm.1058 11747097 PMC6871862

[38] Gaspar M, Welke B, Seehaus F, Hurschler C, Schwarze M. Dynamic Time Warping compared to established methods for validation of musculoskeletal models. Journal of Biomechanics. 2017 Apr;55:156–61. doi: 10.1016/j.jbiomech.2017.02.025 28285744

[39] Lima FT, Souza VMA. A large comparison of normalization methods on time series. Big Data Research. 2023 Nov 28;34:100407. doi: 10.1016/j.bdr.2023.100407

[40] Murakami T, Murray K. Outcomes of knee disarticulation and the influence of surgical techniques in dysvascular patients: A systematic review. Prosthet Orthot Int. 2016 Aug;40(4):423–35. doi: 10.1177/0309364615574163 25820640

[41] Polfer EM, Hoyt BW, Bevevino AJ, Forsberg JA, Potter BK. Knee disarticulations versus transfemoral amputations: functional outcomes. J Orthop Trauma. 2019 Jun;33(6):308–11. doi: 10.1097/BOT.0000000000001440 31124910

[42] Heitzmann DWW, Leboucher J, Block J, Günther M, Putz C, Götze M, et al. The influence of hip muscle strength on gait in individuals with a unilateral transfemoral amputation. PLoS One. 2020;15(9):e0238093. doi: 10.1371/journal.pone.0238093 32877428 PMC7467296

[43] Nakamura T, Mitsumoto A, Okita Y, Maruyama T, Maeno M, Tobimatsu Y. MRI analysis of the soft tissues of the residual limbs in persons with transfemoral amputation. JPSO. 2019;35(3):212–8. doi: 10.11267/jspo.35.212

[44] Chen Z, Franklin DW. Musculotendon parameters in lower limb models: Simplifications, uncertainties, and muscle force estimation sensitivity. Ann Biomed Eng. 2023 Jun;51(6):1147–64. doi: 10.1007/s10439-023-03166-5 36913088 PMC10172227

[45] Santos GF, Gomes AA, Sacco ICN, Ackermann M. Predictive simulation of diabetic gait: individual contribution of ankle stiffness and muscle weakening. Gait Posture. 2017 Oct;58:208–13. doi: 10.1016/j.gaitpost.2017.07.124 28806708

[46] Nene A, Byrne C, Hermens H. Is rectus femoris really a part of quadriceps? Assessment of rectus femoris function during gait in able-bodied adults. Gait Posture. 2004 Aug;20(1):1–13. doi: 10.1016/S0966-6362(03)00074-2 15196513

[47] Robinson MA, Vanrenterghem J, Pataky TC. Statistical Parametric Mapping (SPM) for alpha-based statistical analyses of multi-muscle EMG time-series. J Electromyogr Kinesiol. 2015 Feb;25(1):14–9. doi: 10.1016/j.jelekin.2014.10.018 25465983

[48] Benton AM, Amiri P, Henson DP, Sivapuratharasu B, Mcgregor AH, Bull AMJ. Characterization of muscle recruitment during gait of bilateral transfemoral and through-knee persons with limb loss. Front Bioeng Biotechnol. 2023;11:1128528. doi: 10.3389/fbioe.2023.1128528 37082215 PMC10110921

[49] Wentink EC, Prinsen EC, Rietman JS, Veltink PH. Comparison of muscle activity patterns of transfemoral amputees and control subjects during walking. J NeuroEngineering Rehabil. 2013;10(1):87. doi: 10.1186/1743-0003-10-87 23914785 PMC3750514

