# Supplementary material for: Predicting prosthetic gait and the effects of induced stiff-knee gait
Source: PLoS One. 2025 Jan 2;20(1):e0314758. doi: 10.1371/journal.pone.0314758 (PMC11695016; doi:10.1371/journal.pone.0314758)
Supplement: S1 File — File containing additional tables and figures. (PDF) [file pone.0314758.s001.pdf]

Supporting Information: Predicting prosthetic gait and the effects of induced stiff-knee gait

Gilmar F. Santos[1*], Eike Jakubowitz[1], Christof Hurschler[1]

[1] Hannover Medical School, Laboratory for Biomechanics and Biomaterials, Department of Orthopedic Surgery, DIAKOVERE Annastift, Hannover, Germany

* Corresponding author
E-mail: FernandesdosSantos.Gilmar@mh-hannover.de

Abstract

Prosthetic gait differs considerably from the unimpaired gait. Studying alterations in the gait patterns could help to understand different adaptation mechanisms adopted by these populations. This study investigated the effects of induced stiff-knee gait (SKG) on prosthetic and healthy gait patterns and the capabilities of predictive simulation. Self-selected speed gait of two participants was measured: one healthy subject and one knee disarticulation subject using a variable-damping microprocessor controlled knee prosthesis. Both performed unperturbed gait and gait with restricted knee flexion. Experimental joint angles and moments were computed using OpenSim and muscle activity was measured using surface electromyography (EMG). The differences between the conditions were analyzed using statistical parametric mapping (SPM). Predictive models based on optimal control were created to represent the participants. Additionally, a hypothetical unimpaired predictive model with the same anthropometric characteristics as the amputee was created. Some patterns observed in the experimental prosthetic gait were predicted by the models, including increased knee flexion moment on the contralateral side caused by SKG in both participants, which was statistically significant according to SPM. With the exception of the rectus femoris muscle, we also found overall good agreement between measured EMG and predicted muscle activation. We predicted more alterations in activation of the hip flexors than other muscle groups due to the amputation and in the activation of the biceps femoris short head, quadratus femoris, and tibialis anterior due to SKG. In summary, we demonstrated that the method applied in this study could predict gait alterations due to amputation of the lower limb or due to imposed SKG.

1 Supplementary Figures

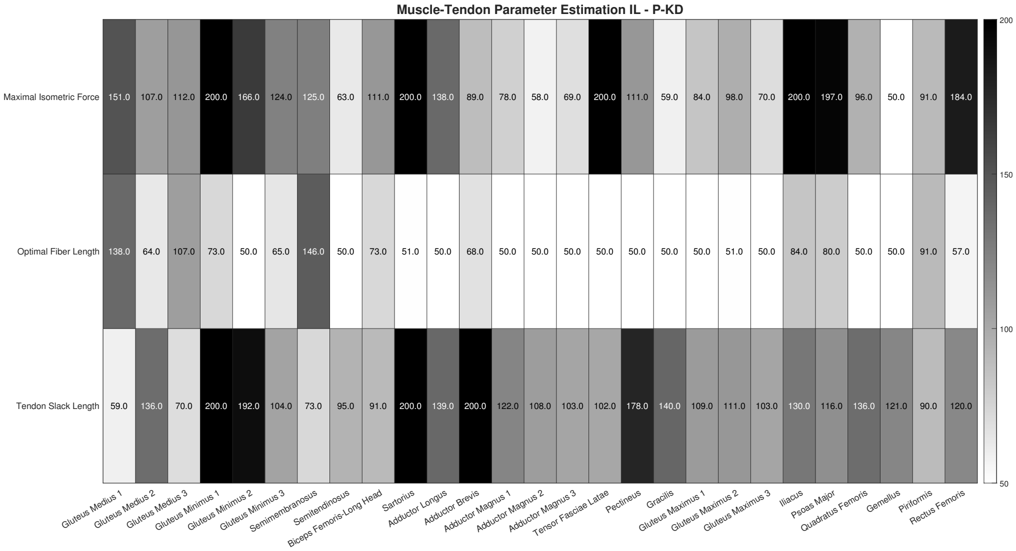

Fig S1. Heat map of the muscle tendon parameter estimation. Result of the muscle tendon parameter estimation on ipsilateral (IL) side of P-KD as a percentage of change relative to generic value. The limits were 50% and 200%, which represented half and double of the generic values, respectively.

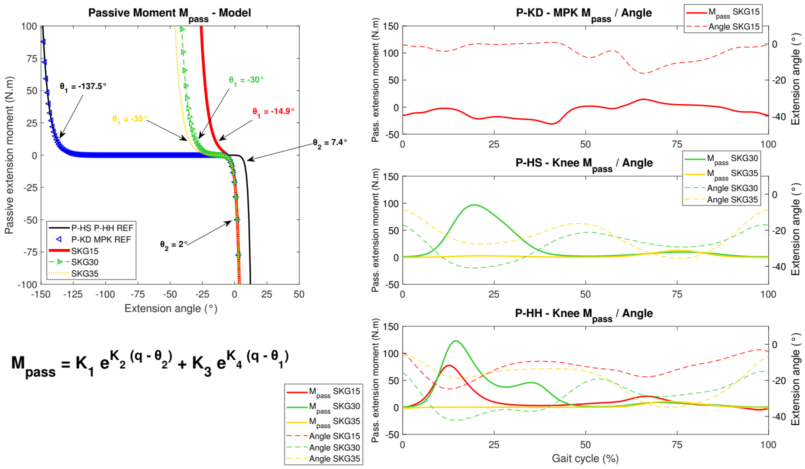

Fig S2. Joint passive moment. MPK and knee sagittal passive moment vs. sagittal angle of the model, equation of joint passive moment, MPK and knee sagittal passive moment and sagittal angle during the gait cycle of the predictions (P-KD, P-HS, and P-HH) for the REF and SKG conditions.

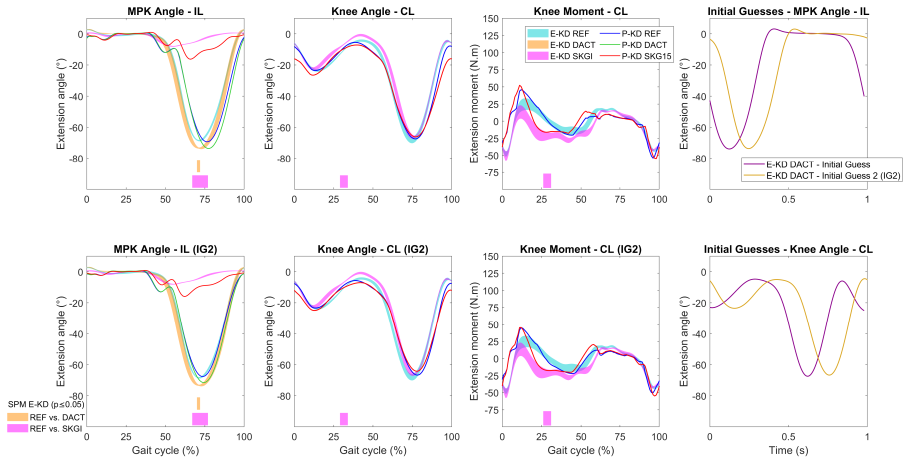

Fig S3. Kinematics and kinetics of the knee disarticulation subject using different initial guesses. Ipsilateral (IL) MPK sagittal angle, and contralateral (CL) knee sagittal angle and moment of P-KD unsing different initial guesses (IG2), and SPM analysis of E-KD for REF vs. DACT and REF vs. SKGI (bars below the graphs).

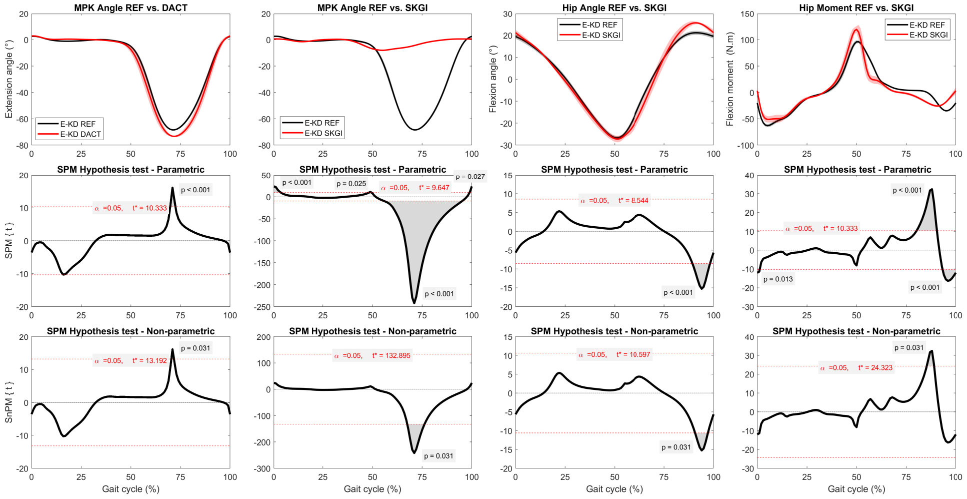

Fig S4. SPM analysis of kinematics and kinetics on ipsilateral (IL) side of the knee disarticulation subject. MPK sagittal angle, and hip sagittal angle and moment for E-KD (top panels), and SPM analysis of E-KD for REF vs. DACT and REF vs. SKGI (bottom panels).

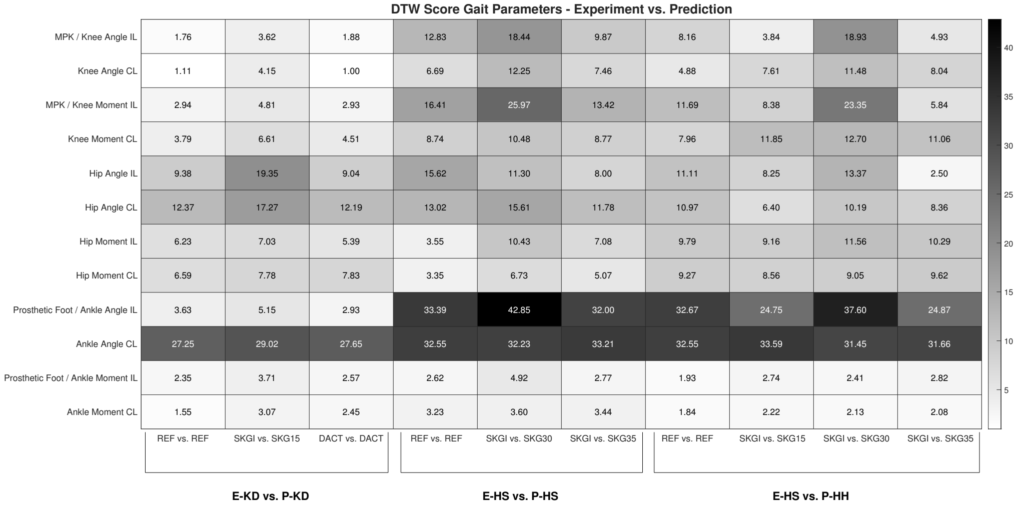

Fig S5. Heat map of DTW scores of the similarity in kinematics and kinetics. Results of the normalized MPK, knee, hip, prosthetic foot, and ankle sagittal angles and moments comparing experiment vs. prediction for different models and conditions. Smaller (lighter) DTW values indicate greater agreement; higher (darker) DTW values indicate lesser agreement between the curves.

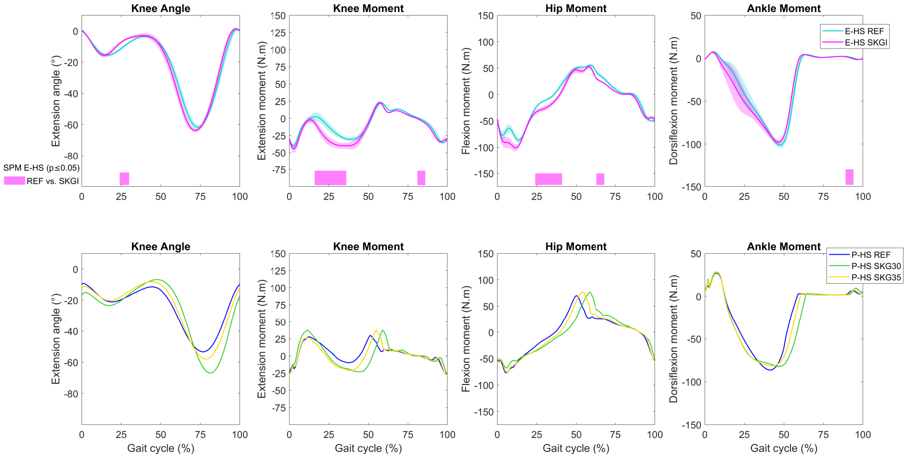

Fig S6. Kinematics and kinetics on contralateral (CL) side of the healthy subject. Knee sagittal angle and moment and hip and ankle sagittal moments for E-HS (top panels) and P-HS (bottom panels), and SPM analysis of E-HS for REF vs. SKGI (bars below the graphs). Corresponding ipsilateral curves are presented in Fig 5 (main manuscript).

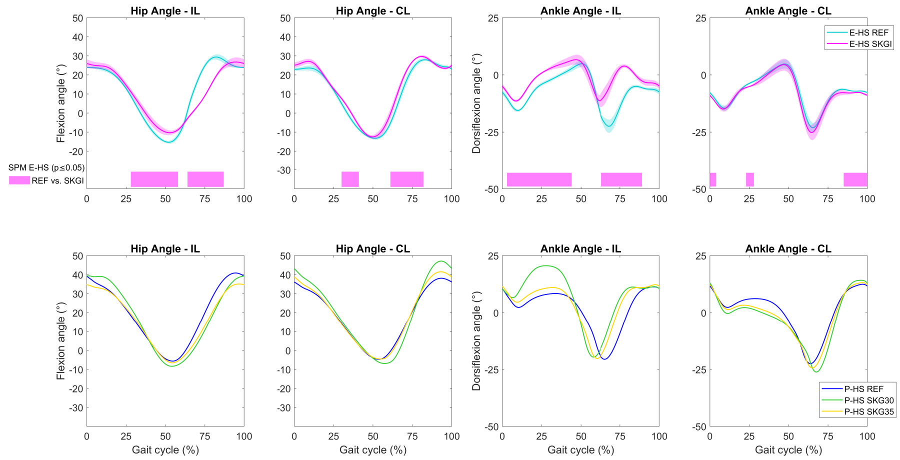

Fig S7. Kinematics of the healthy subject. Hip and ankle sagittal angles for E-HS (top panels) and P-HS (bottom panels) on ipsilateral (IL) and contralateral (CL) sides, and SPM analysis of E-HS for REF vs. SKGI (bars below the graphs).

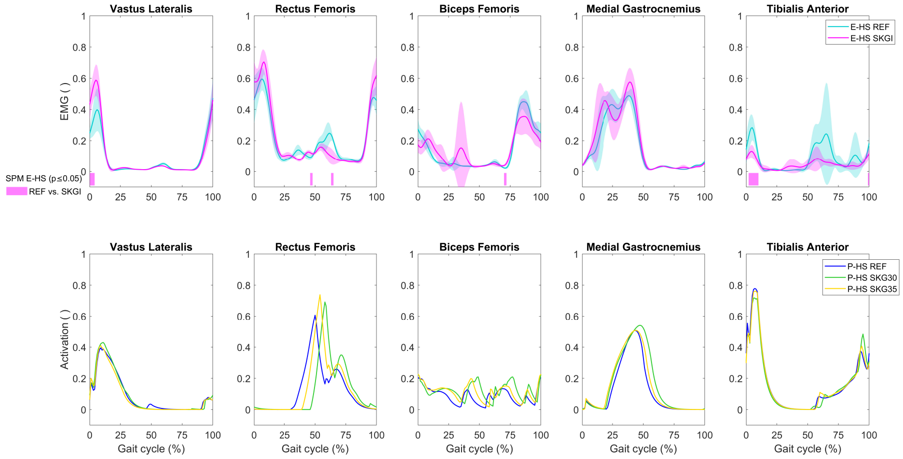

Fig S8. EMG and muscle activation on contralateral (CL) side of the healthy subject. EMG for E-HS (top panels), muscle activation for P-HS (bottom panels), and SPM analysis of E-HS for REF vs. SKGI (bar below the graph). Corresponding ipsilateral curves are presented in Fig 6 (main manuscript). The predicted muscle activation curves depicted in the graph of biceps femoris are composed of the maximal values between biceps femoris long and short heads.

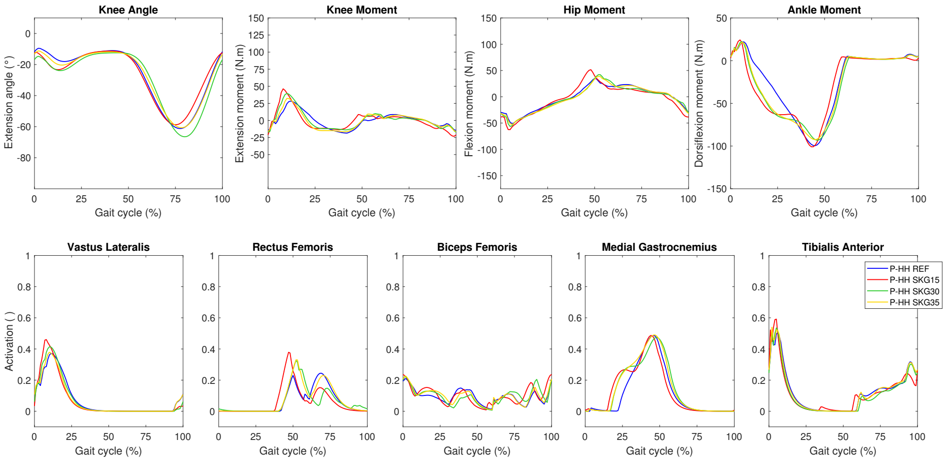

Fig S9. Kinematics, kinetics and muscle activation on contralateral (CL) side of the hypothetical healthy predictive model of the amputee. Knee sagittal angle and moment, hip and ankle sagittal moments (top panels), and muscle activation (bottom panels) for P-HH. Corresponding ipsilateral curves are presented in Fig 7 (main manuscript). The predicted muscle activation curves depicted in the graph of biceps femoris are composed of the maximal values between biceps femoris long and short heads.

2 Supplementary Tables

Table S1. Weight factors of the objective function of gait predictive simulations presented in Eq. 3 (main manuscript).

**Predictive model**	W_{P1}	W_{P2}	W_{P3}	W_{P4}	W_{P5}
P-KD	10000	100	20000	0.001	100
P-HH	10000	100	20000	0.001	–
P-HS	5000	100	50000	0.001	–

Table S2. Number of iterations, wall time, objective cost, and stride time of gait predictive simulations.

**Predictive model**	**Condition**	**Number of iterations**	**Wall time (h)**	**Objective cost**	**Stride time (s)**
P-KD	REF	677	1.33	176.94	1.10
	REF (IG2)	821	3.39	190.56	1.13
	DACT	654	2.80	178.01	1.12
	DACT (IG2)	888	4.37	190.46	1.15
	SKG15	1733	8.30	193.04	1.06
	SKG15 (IG2)	1111	4.34	198.18	1.09
P-HH	REF	806	4.45	150.72	1.01
	SKG15	1964	9.17	171.92	1.10
	SKG30	893	3.40	154.45	1.12
	SKG35	920	5.11	159.56	1.03
P-HS	REF	2606	13.23	162.97	1.03
	SKG30	1423	9.11	178.49	1.10
	SKG35	2834	11.92	171.62	1.04

Table S3. Muscles abbreviation, full name and functional group.

**Abbreviation**	**Muscle full name**	**Functional group**
glut med1	Gluteus Medius 1	Hip abduction, flexion, internal rotation
glut med2	Gluteus Medius 2	Hip abduction
glut med3	Gluteus Medius 3	Hip abduction, extension, external rotation
glut min1	Gluteus Minimus 1	Hip abduction, flexion, internal rotation
glut min2	Gluteus Minimus 2	Hip abduction
glut min3	Gluteus Minimus 3	Hip abduction, extension, external rotation
semimem	Semimembranosus	Hip adduction, extension; knee flexion
semiten	Semitendinosus	Hip adduction, extension; knee flexion
bifemlh	Biceps Femoris Long Head	Hip adduction, extension; knee flexion
bifemsh	Biceps Femoris Short Head	Knee flexion
sar	Sartorius	Hip abduction, flexion; knee flexion
add long	Adductor Longus	Hip adduction, flexion
add brev	Adductor Brevis	Hip adduction, flexion
add mag1	Adductor Magnus 1	Hip adduction, extension
add mag2	Adductor Magnus 2	Hip adduction, extension
add mag3	Adductor Magnus 3	Hip adduction, extension
tfl	Tensor Fasciae Latae	Hip abduction, flexion, internal rotation
pect	Pectineus	Hip adduction, flexion
grac	Gracilis	Hip adduction, flexion; knee flexion
glut max1	Gluteus Maximus 1	Hip abduction, extension
glut max2	Gluteus Maximus 2	Hip extension
glut max3	Gluteus Maximus 3	Hip extension
iliacus	Iliacus	Hip flexion, internal rotation
psoas	Psoas Major	Hip flexion, internal rotation
quad fem	Quadratus Femoris	Hip external rotation
gem	Gemellus	Hip external rotation
peri	Piriformis	Hip abduction, external rotation
rect fem	Rectus Femoris	Hip flexion; knee extension
vas med	Vastus Medialis	Knee extension
vas int	Vastus Intermedius	Knee extension
vas lat	Vastus Lateralis	Knee extension
med gas	Medial Gastrocnemius	Knee flexion; ankle plantarflexion
lat gas	Lateral Gastrocnemius	Knee flexion; ankle plantarflexion
soleus	Soleus	Ankle plantarflexion
tib post	Tibialis Posterior	Ankle plantarflexion; foot inversion
flex dig	Flexor Digitorum Longus	Ankle plantarflexion; foot inversion
flex hal	Flexor Hallucis Longus	Ankle plantarflexion; foot inversion
tib ant	Tibialis Anterior	Ankle dorsiflexion; foot inversion
per brev	Peroneus Brevis	Ankle plantarflexion; foot eversion
per long	Peroneus Longus	Ankle plantarflexion; foot eversion
per tert	Peroneus Tertius	Ankle dorsiflexion; foot eversion
ext dig	Extensor Digitorum Longus	Ankle dorsiflexion; foot eversion
ext hal	Extensor Hallucis Longus	Ankle dorsiflexion; foot inversion
ercspn	Erector Spinae	Lumbar
intobl	Internal Oblique	Lumbar
extobl	External Oblique	Lumbar
